# Constitutive activity of metabotropic glutamate receptor 7

**DOI:** 10.1186/s12868-015-0154-6

**Published:** 2015-03-24

**Authors:** Paul J Kammermeier

**Affiliations:** Department of Pharmacology and Physiology, University of Rochester Medical Center, 601 Elmwood Ave, Box 711, Rochester, NY 14642 USA

**Keywords:** Metabotropic glutamate receptor, Calcium channel, Sympathetic neuron

## Abstract

**Background:**

Metabotropic glutamate receptors (mGluRs) are class C G protein coupled receptors with widespread central nervous system expression. mGluR7 is a member of this family that has been implicated in numerous physiological and pathological processes, but the very low potency of mGluR7 for glutamate, its natural ligand, raise questions about the nature of its physiological role.

**Results:**

Here, evidence is presented using heterologous expression in sympathetic neurons from the rat superior cervical ganglion (SCG) and modulation of the native SCG calcium currents as an assay for receptor signaling, that mGluR7 exhibits constitutive activity. This activity is detectable as basal calcium channel modulation in the absence of ligand that is not observed in untransfected cells or those transfected with other members of the mGluR family. Further, this basal channel modulation was reversibly inhibited with the mGluR7 inverse agonist MMPIP. Surprisingly, MMPIP did not strongly inhibit agonist-induced mGluR7 activation. Finally, the selective mGluR8 agonist (R,S)-PPG was also able to act as an inverse agonist at mGluR7.

**Conclusions:**

These findings introduce a novel potential physiological role for mGluR7 in the nervous system, that of a constitutively active receptor, and thereby suggest a model in which mGluR7 signaling may be impactful without the need to invoke strong receptor activation by millimolar concentrations of extracellular glutamate. Constitutive activity of mGluR7 may be eliminated or reduced by the presence of other group III mGluRs, perhaps due to heterodimer formation. In addition, both MMPIP and PPG acted as inverse agonists at mGluR7, and agonists at mGluR8.

## Background

Metabotropic glutamate receptors (mGluRs) are class C G protein coupled receptors with widespread expression in the mammalian nervous system [1]. As such, mGluRs are involved in many neural processes regulating important physiological and pathological processes. Compared with many G protein coupled receptor subtypes, mGluRs have relatively low affinity/potency for their native ligand, glutamate [2]. Most mGluRs exhibit K_D_ or EC_50_ values from the low to mid micromolar range [3]. This is likely the case because basal extracellular glutamate levels in the nervous system tend to be relatively high [4,5]. The group III mGluR, mGluR7 exhibits the lowest potency of any mGluR, with estimates in the hundreds of micromolar to low millimolar range, with full activation requiring nearly 10 mM glutamate [6]. Thus, it is difficult to understand the physiological role of a receptor that may only rarely get fully activated. Here evidence is presented that when mGluR7 is expressed in neurons, it shows a detectable level of constitutive activity. This activity appeared to be relatively low compared to full activation of the receptor, and was reduced when other group III mGluRs were coexpressed. It was further demonstrated that mGluR7 constitutive signaling can be inhibited by the selective mGluR7 antagonist MMPIP [7], and also by the mGluR8 selective agonist PPG [8].

## Methods

### SCG neuron isolation, cDNA injection, and plasmids

The neuronal isolation and injection procedures have been previously described [9]. Briefly, SCG were dissected from adult Wistar rats and incubated in Earle’s balanced salt solution (Life Technologies, Rochelle, MD) with 0.55 mg/ml trypsin (Worthington, Freehold, NJ), 1.6 mg/ml Type IV collagenase (Worthington) for 1 hour at 35°C. Cells were then spun twice, transferred to minimum essential medium (Fisher Scientific, Pittsburgh, PA), plated, and incubated at 37°C until cDNA injection. cDNA injections was performed with an Eppendorf 5247 microinjector and Injectman NI2 micromanipulator (Madison, WI) 4–6 hours following cell isolation. Plasmids were stored at −20°C as a 1–2 μg/μl stock solution in TE buffer (10 mM TRIS, 1 mM EDTA, pH 8). The mGluR7, 8, and 4 clones (in pCDNA3.1+) were obtained from cDNA.org (Missouri S&T cDNA Resource Center, Rolla, MO). Concentrations of cDNAs injected were as indicated in the text. All neurons were co-injected with green fluorescent protein cDNA (0.02 μg/μl; pEGFPC1; Clontech Laboratories, Palo Alto, CA, USA) for identification of expressing cells. Cells were the incubated overnight at 37°C and experiments are performed the following day. All animal protocols were approved by the University of Rochester’s Committee on Animal Resources (UCAR).

### Electrophysiology and data analysis

Patch-clamp recordings were made using 8250 glass (King Precision Glass, Claremont, CA). Pipette resistances were 0.8-3 MΩ yielding uncompensated series resistances of 1–5 MΩ. Series resistance compensation of ≥ 80% was used in all recordings. Data was recorded using an Axopatch 1D patch-clamp amplifier from Axon (now Molecular Devices, Sunnyvale, CA). Voltage protocol generation and data acquisition were performed using custom procedures written for the Igor Pro software software package (Wavemetrics, Lake Oswego, OR) by Stephen R. Ikeda (NIH, NIAAA) on a MacMini Intel DuoCore computer with an Instrutech ITC18 data acquisition board (HEKA Elektronik). Currents were sampled at 100 kHz low-pass filtered at 5 kHz, digitized, and stored on the computer for later analysis. All patch-clamp experiments were performed at 21–24°C (room temperature). Data analysis was performed using Igor Pro software (WaveMetrics, Lake Oswego, OR). The external (bath) calcium current recording solution contained (in mM):145 tetraethylammonium (TEA) methanesulfonate (MS),10 4-(2-Hydroxyethyl)-1-piperazineethanesulfonic acid (HEPES), 15 glucose, 10 CaCl_2_, and 300 nM tetrodotoxin, pH 7.4, osmolality 320 mOsm/kg. The internal (pipette) solution contained: 120 N-methyl-Dglucamine (NMG) MS, 20 TEA, 11 EGTA, 10 HEPES, 10 sucrose, 1 CaCl_2_, 4 MgATP, 0.3 Na_2_GTP, and 14 tris-creatine phosphate, pH 7.2, osmolality 300 mOsm/kg. L-AP4, 6-(4-Methoxyphenyl)-5-methyl-3-(4-pyridinyl)-isoxazolo[4,5-*c*]pyridin-4(5*H*)-one hydrochloride (MMPIP), and (*RS*)-4-Phosphonophenylglycine (PPG) were obtained from Tocris Bioscience (R&D Systems, Bristol, UK).

## Results

### Modulation of SCG calcium currents by mGluR7

**Figure 1:**
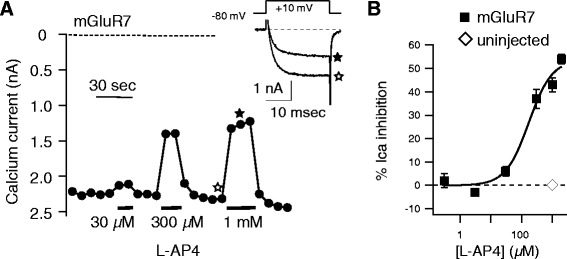
**mGluR7 expression in rat SCG neurons. A**, Time course of calcium current amplitude during a test pulse to +10 mV (measured at 10 msec after the start of the step) in a rat SCG neuron expressing mGluR7. Application of 30 μM, 300 μM, and 1 mM L-AP4 at the indicated times produced a rapid and reversible inhibition of the current. Sample control (☆) and inhibited (★) current traces are shown in the *inset*. **B**, Average responses (±SEM) to a range of [L-AP4] from neurons expressing mGluR7 (■), and to 1 mM L-AP4 in control, uninjected cells (♢). Solid line represents a fit to the Hill equation, revealing an IC_50_ value of approximately 170 μM.

To examine its signaling, mGluR7 cDNA was injected into nuclei of isolated SCG neurons from the adult rat along with EGFP to identify successfully injected cells [9]. The following day, whole-cell patch-clamp recordings were made in expressing cells under conditions designed to isolate currents through the native, mostly N-type [10], calcium channels. Because mGluR7 couples to the G_i/o_ family [3], calcium current was expected to be robust and mediated by Gβγ subunits upon receptor activation. Indeed, application of the group III mGluR selective agonist L-AP4 (which activates mGluR4, 6, 7, & 8 [3]) produced a strong, reversible inhibition of the SCG calcium currents (Figure 1). Sample control and 1 mM L-AP4 inhibited current traces obtained during a 25 msec test pulse to +10 mV are shown in the inset of Figure 1A. As illustrated by the example cell shown in Figure 1A as well as the average L-AP4 dose–response curve in Figure 1B (squares), mGluR7 responded with relatively low potency to this agonist, exhibiting an apparent IC_50_ of about 170 μM. This is about 10 fold lower potency than that observed with other group III mGluRs [3]. Activation of mGluR7 by glutamate, the natural ligand also requires considerably more agonist than other mGluRs [3], suggesting that mGluR7 is a low potency receptor in general. It should be noted that the response did not reach saturation even at 2 mM L-AP4. However at higher concentrations, L-AP4 was not soluble. The response of uninjected SCG neurons to 1 mM L-AP4 is also shown in Figure 1B (open diamond, those cells were inhibited 0 ± 0.4%, n = 5). These cells showed no detectable response to L-AP4, indicating that the responses observed were due to heterologously expressed mGluR7.

### mGluR7 exhibits constitutive activity

The mechanism of calcium current inhibition in rat SCG neurons by G_i/o_ coupled receptors, such as mGluR7, is via direct interaction of active Gβγ with the channel’s pore forming subunit [11-13]. Bean first characterized this modulation by proposing a model in which uninhibited channels gate in a “willing” mode that allows channel opening at moderate voltages [14]. Inhibition causes the channels to enter a “reluctant” gating mode that requires stronger depolarization to open the channels. These mechanistic features give rise to a few unique characteristics. The first is the appearance of a slow activation phase at moderate membrane potentials evident in the sample current trace in Figure 1 (*inset*). The second is a temporary relief of inhibition following strong depolarizations. Both of these features are diagnostic hallmarks of Gβγ-mediated calcium channel inhibition, and both are thought to result at least in part from the physical dissociation of Gβγ from the channels at more depolarized potentials [15]. Finally, modulation of each member of the CaV2.x family has been shown to occur through this pathway [16,17].

**Figure 2:**
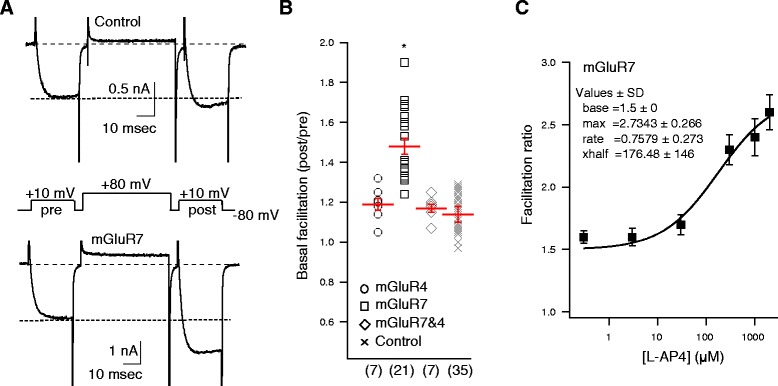
**Calcium current basal facilitation is elevated in SCG neurons expressing mGluR7. A**. Sample calcium current traces from a control cell (*upper*) and an mGluR7 expressing cell (*lower*) obtained using the triple-pulse voltage protocol (shown in *center*). Each current trace was obtained in the absence of any receptor agonist. Note that the post/pre ratio is elevated in the mGluR7 expressing SCG neuron, reflecting the higher basal facilitation levels in those cells. **B**, Average (± SEM) basal facilitation, and values from individual cells, illustrating the facilitation values from SCG neurons expressing mGluR4 (°), mGluR7 (□), mGluR4 and 7 together (♢), and in control SCG neurons (✕); uninjected and expressing other mGluRs recorded on separate days from the cells in the other groups). Total number of cells in each group is shown in parentheses. **C**, L-AP4 concentration-response curve from mGluR7 expressing SCG neurons expressed as facilitation (post/pre) values. Solid line represents a fit to the Hill equation, with baseline fixed at 1.5. Fits to free parameters are as indicated (*inset*).

By employing a triple-pulse voltage protocol [18] (Figure 2A), the degree to which this pathway is active can be monitored in real time. This protocol consists of two 25 msec test pulses to +10 mV. The first (the prepulse, or “pre” in Figure 2A) precedes a 45 msec conditioning pulse to +80 mV, and the other (the postpulse, or “post” in Figure 2A) follows it after a brief, 5 msec step back to −80 mV. Since the conditioning pulse can transiently relieve Gβγ inhibition, the facilitation ratio (post/pre) provides a real time measure of the degree to which this pathway is active. This approach has the added advantage of allowing an objective measure of the Gβγ pathway even in the absence of receptor agonists. For example, higher levels of constitutive receptor activity will produce more free Gβγ, which produces a greater inhibition and thus a higher facilitation ratio.

Interestingly, SCG neurons made to express mGluR7 exhibited a significantly higher basal facilitation ratio (prior to application of any agonist) than neurons expressing mGluR4 and recorded on the same days (Figure 2A, B). Cells expressing mGluR7 had a basal facilitation ratio of 1.48 ± 0.04 (n = 21), compared with 1.19 ± 0.03 (n = 7) cells expressing mGluR4, and recorded on the same days. These data suggest that mGluR7 exhibits some degree of constitutive signaling in the absence of agonist which results in a higher than average amount of free Gβγ.

The basal facilitation value of the mGluR4 expressing cells was indistinguishable from a larger group of control cells obtained on separate days (within one week of the data described above), including uninjected cells and those expressing mGluR2, mGluR4 or both receptors together. The pooled results from these cells are shown in Figure 2A and B, labeled “Control.” The average basal facilitation of these control neurons was 1.14 ± 0.04 (n = 35). Interestingly, when mGluR7 and 4 were co-expressed in the same neurons, the basal facilitation resembled control cells at 1.17 ± 0.02 (n = 7), suggesting that constitutive signaling of mGluR7 is inhibited by the presence of mGluR4, although the mechanism of this phenomenon is not known.

Next, the degree to which mGluR7 signaled constitutively was investigated. While basal facilitation was elevated in mGluR7 expressing cells, the facilitation ratio in these neurons was not high compared with that seen in cells over-expressing Gβγ [11,19], or in the presence of saturating concentrations of agonists of G_i/o_ coupled receptors [20]. Indeed, when the facilitation ratio that resulted from application of various concentrations of L-AP4 was plotted, it can be seen that this value could be increased to over 2.5 by even the somewhat sub-saturating concentrations of 300 μM - 2 mM L-AP4 (Figure 2C). Thus, it can be concluded that while mGluR7 does appear to signal constitutively, the degree of activation is relatively subtle compared with full activation of the receptor.

### Inhibition of mGluR7 constitutive activity with an inverse agonist

**Figure 3:**
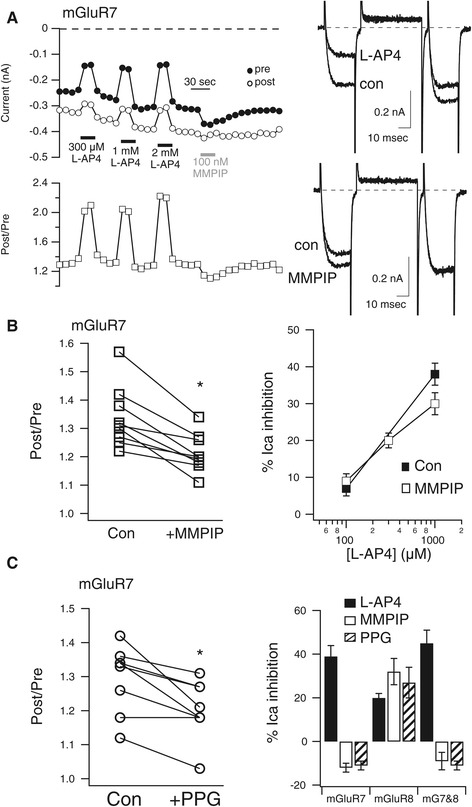
**mGluR7 is a constitutively active receptor. A**, Time course of calcium current amplitudes (*upper left*) and facilitation ratios (*lower left*) from the “pre” and “post” test pulses obtained with the triple pulse protocol in a representative SCG neuron during application of the indicated concentrations of L-AP4 and 100 nM MMPIP. Sample current traces from the same cell are shown to the *right*. Control and L-AP4 inhibited currents are shown (*upper right*), as are control and MMPIP enhanced currents (*lower right*). **B**, Plot of control facilitation values and those in the presence of MMPIP (*left*) in mGluR7 expressing neurons, paired from each cell. * indicates significant difference (p < 0.05, paired T-test). L-AP4 concentration-response curve is also shown for two [L-AP4]s when applied alone (■), and three [L-AP4]s when applied in the presence of 100 nM MMPIP (□). No significant differences were detected. **C**, Plot of control facilitation values and those in the presence of PPG (*left*) in mGluR7 expressing neurons, paired from each cell. *indicates significant difference (p < 0.05, paired T-test). Average responses (±SEM) to L-AP4 (black bars), MMPIP (white bars), and PPG (striped bars) in SCG neurons expressing mGluR7, mGluR8, and mGluR7&8 together, as indicated.

If the elevated basal facilitation observed in mGluR7 expressing sympathetic neurons was in fact due to elevated mGluR7 signaling in the absence of agonist, then the facilitation should be reduced and current should be slightly enhanced upon application of an antagonist with inverse agonist properties. To test this prediction, the mGluR7 selective antagonist 6-(4-Methoxyphenyl)-5-methyl-3-(4-pyridinyl)-isoxazolo[4,5-*c*]pyridin-4(5*H*)-one hydrochloride (MMPIP) was used [7]. As shown in the sample cell in Figure 3A, application of 100 nM MMPIP to an mGluR7 expressing SCG neuron did in fact both transiently enhance the basal current level and reduce facilitation, consistent with a reduction in constitutive signaling of the receptors in that cell. Sample current traces (*right*) show control and L-AP4 inhibited currents using the triple pulse voltage protocol described above (*upper*) and control and MMPIP enhanced current traces (*lower*) from the same cell. Note that while MMPIP enhanced the current in the prepulse, it did not detectably alter the postpulse current, thereby resulting in a smaller post/pre ratio. Figure 3B (*left*) shows post/pre ratio values for all of the mGluR7 expressing cells in which 100 nM MPPIP was applied. In these cells, MMPIP significantly reduced the facilitation ratio from 1.34 ± 0.04 to 1.21 ± 0.02 (n = 9). The absolute magnitude of the prepulse current was also enhanced in these cells from −1.11 ± .22 nA to −1.22 ± 0.25 nA. By contrast, when 100 nM MMPIP was applied to control, uninjected SCG neurons, the current amplitude was not significantly enhanced and the facilitation ratio was not significantly reduced. Current amplitude of control cells was −1.06 ± .1 nA in control and −1.01 ± .11 nA in MMPIP. The facilitation ratio was 1.39 ± 0.05 and 1.42 ± 0.05 in control and MMPIP, respectively (n = 4), suggesting that the effects of MMPIP were due to the expression of mGluR7.

Interestingly, while MMPIP appeared to effectively reduce constitutive mGluR7 activity, the same concentration of MMPIP did not effectively inhibit L-AP4 activation of the receptor in the 100 μM - 1 mM range (Figure 3B, *right*). In SCG neurons expressing mGluR7, 100 μM and 1 mM L-AP4 were applied and washed off to obtain control agonist responses (Figure 3B, *right*, solid squares) In the same cells, 100 μM, 300 μM, and 1 mM L-AP4 were applied in the presence of 100 nM MMPIP (Figure 3B, *right*, open squares). Responses in the presence of MMPIP were not significantly reduced compared to control responses. This result was in contrast with those reported by Suzuki et al. [7], in which 100 nM MMPIP was effective as both an inverse agonist and in inhibiting L-AP4 responses. The reason for this discrepancy is unclear, but in this study the human mGluR7 clone was transiently expressed in rat SCG neurons, while in that study, rat mGluR7 was stably expressed in Chinese Hamster Ovary (CHO) cells. It is possible that affinity of MMPIP for human mGluR7 is slightly lower than for the rat sequence. Unfortunately, application of higher concentrations of MMPIP in the SCG calcium current assay was problematic due to solubility issues and direct effects of the solvent (DMSO) on calcium currents.

### Effect of an mGluR8 selective agonist on mGluR7

Fortuitously, the mGluR8 selective agonist (*RS*)-4-Phosphonophenylglycine (PPG) [8] was also applied to SCG neurons expressing mGluR7. Similar to the effect of MMPIP, 1 μM PPG also significantly reduced the facilitation ratio in mGluR7 expressing cells, suggesting that it may also act as an inverse agonist at these receptors (Figure 3C, *left*). In mGluR7 expressing SCG neurons, application of 1 μM PPG reduced the facilitation ratio from 1.35 ± 0.06 to 1.27 ± 0.04 (n = 6). As a positive control, PPG was also applied to SCG neurons expressing mGluR8 (Figure 3C, *right*). In these cells, the group III mGluR agonist L-AP4 (1 mM) produced a 20 ± 2% inhibition of the calcium current (n = 3). The current was inhibited similarly, 27 ± 7%, by 1 μM PPG in the same cells, confirming the agonistic activity of PPG. Interestingly, in the same cells, application of 100 nM MMPIP also appeared to function as an agonist, as it induced a reversible inhibition of the calcium current of 32 ± 6%. Thus despite their structural dissimilarity, both MMPIP and PPG appear to act as inverse agonists at mGluR7 and agonists at mGluR8, two receptors with relatively high homology.

Finally, 1 mM L-AP4, 100 nM MMPIP, and 1 μM PPG were applied to SCG neurons co-expressing both mGluR7 and mGluR8 (Figure 3C, *right*). It should first be noted that these neurons did not exhibit elevated basal facilitation as the cells expressing mGluR7 alone did. The facilitation ratio in mGluR7/8 expressing neurons was 1.25 ± 0.02 (n = 5), consistent with the observation (Figure 2, above) that co-expression of mGluR4 with mGluR7 eliminated constitutive activity. However, in the mGluR7/8 expressing neurons, both MMPIP and PPG had effects similar to those in cells expressing mGluR7 alone. That is, each slightly enhanced the current amplitude by 9 ± 4% (Figure 3C, *right*; n = 4), suggesting that while the basal facilitation ratio was not strongly elevated, some constitutive signaling may have been occurring. Indeed, the facilitation ratio was reduced in these cells from 1.25 ± 0.02 to 1.16 ± 0.04 by 100 nM MMPIP and from 1.27 ± 0.03 to 1.19 ± 0.03 by 1 μM PPG. Together, these data indicate that the mGluR8 selective agonist PPG may act as an inverse agonist on mGluR7, and that the mGluR7 inverse agonist can act as an agonist at mGluR8.

## Discussion

In the current study, the signaling properties of mGluR7 were examined adult rat sympathetic neurons from the SCG. Group III mGluRs like mGluR7 couple to G_i/o_ proteins, which mediate a strong, reversible inhibition of Ca_V2_ channels [15,16] mediated by Gβγ [11,12]. Because Gβγ-mediated calcium current inhibition exhibits a characteristic voltage dependence [14,21], assessment of active G protein levels in the presence and absence of receptor ligand is possible using a simple ‘triple-pulse’ voltage protocol [18]. Using this strategy, it was shown that SCG neurons expressing mGluR7 by intranuclear cDNA injection exhibited significantly higher than normal levels of free, active Gβγ, suggesting that expressed mGluR7 may be constitutively signaling. Similar results were not observed in control, uninjected neurons, nor in neurons expressing other mGluR subtypes. Thus suggests that the elevated levels of apparent mGluR7 activation were not likely due to elevated levels of glutamate in the extracellular environment, as the potency of mGluR7 for glutamate is considerably lower than every other mGluR subtype [3]. The degree of constitutive activation of mGluR7 was limited however, since application of the agonist L-AP4 consistently further activated the receptor, and produced a greater level of calcium current inhibition than was observed in the absence of ligand.

The suggestion that mGluR7 signals constitutively was confirmed by application of the mGluR7 inverse agonist MMPIP, which consistently increased current amplitudes in mGluR7 expressing cells, but not in uninjected cells. Further, MMPIP significantly reduced the current facilitation ratio, an independent measure of the degree of active Gβγ in the cells. These data suggest that the elevated Gβγ levels in mGluR7 expressing SCG neurons were the result of signaling of mGluR7 at the level of the receptor, and not likely a secondary effect of mGluR7 over-expression.

The observation that mGluR7 can signal constitutively has been posited previously [7], but that observation came only from mGluR7 expressed in CHO cells pretreated with forskolin, a somewhat less physiological system than the one employed here. Indeed, another study [22] showed elevated calcium channel facilitation in cerebellar granule neurons that was inhibited by the mGluR7 binding protein MacMARCKS. However in that study, untransfected neurons had similar channel facilitation as mGluR7 transfected cells, despite the fact that the authors could not detect somatic mGluR7 in untransfected cells. Therefore, the authors could neither confirm nor refute the potential constitutive activity of mGluR7 in those cells. Thus this is the first definitive demonstration of constitutive mGluR7 signaling in neurons.

In addition to the inhibition of mGluR7 constitutive activity by the inverse agonist MMPIP, the effect of the selective mGluR8 agonist PPG was also examined. Surprisingly, PPG showed similar responses when applied as MMPIP, both in inhibiting mGluR7 activity and in its agonist activity at mGluR8, suggesting that these two relatively dissimilar compounds have similar activities at both receptors. It is likely however, that the similar responses induced by these compounds are coincidental because MMPIP binds at least mGluR7 at an allosteric site, while PPG has some structural similarity to glutamate and L-AP4, and likely functions as an orthosteric agonist at mGluR8. MMPIP likely acts on mGluR8 as an allosteric agonist, or possibly as an ago-PAM (an agonist and positive modulator acting at an allosteric site), although there is currently no evidence for PAM activity of MMPIP at mGluR8.

A surprising and as yet little understood phenomenon is the abolishment or reduction of apparent mGluR7 constitutive activity when other group III mGluRs were coexpressed. Expression of mGluR4 with mGluR7 strongly reduced basal facilitation values to control levels, and mGluR8 expression reduced facilitation somewhat as well. One possible explanation might be that mGluR7 forms heterodimers with mGluR4 and 8, and that mGluR7 containing dimers do not exhibit constitutive signaling. While we do not have data to thoroughly address this claim here, there are some hints in the pharmacological data that could lead to at least some informed hypotheses. In Figure 3C, it is clear that both MMPIP and PPG produce similar effects as when mGluR7 is expressed alone, but the reduced basal facilitation (compared to mGluR7 expressing cells) suggests that at least some mGluR8 is expressed. One interpretation is that mGluR7/8 heterodimers are predominantly expressed and that these are affected by MMPIP and PPG similarly to mGluR7 homodimers. However, since MMPIP is an allosteric compound and PPG likely acts at the orthosteric site, this explanation may be unlikely. Perhaps the more likely scenario is that under these conditions, a small number of mGluR7/8 heterodimers are expressed with a reduced (compared with cells expressing mGluR7 alone) number of mGluR7 homodimers. This would explain the reduced facilitation values, the enhancement of current by both drugs, and the lack of a clear agonist effect of the drugs as well. The mechanism of the reduced facilitation ratio when mGluR4 was expressed with mGluR7 also remains to be elucidated.

## Conclusions

The results of this study demonstrate that mGluR7 can signal constitutively (in the absence of agonist) when expressed in neurons. This constitutive signaling appears to be reduced when mGluR7 is coexpressed with another group III mGluR.

### Ethics statement

Human subjects research was not conducted in this study.

## Abbreviations

SCG: Superior Cervical Ganglion
MMPIP: 6-(4-Methoxyphenyl)-5-methyl-3-(4-pyridinyl)-isoxazolo[4,5-*c*]pyridin-4(5*H*)-one hydrochloride
PPG: (*RS*)-4-Phosphonophenylglycine
mGluR: Metabotropic glutamate receptor
